# Supplementary material for: Different influences of moral violation with and without physical impurity on face processing: An event-related potentials study
Source: PLoS One. 2020 Dec 16;15(12):e0243929. doi: 10.1371/journal.pone.0243929 (PMC7743946; doi:10.1371/journal.pone.0243929)
Supplement: S1 Appendix — (DOCX) [file pone.0243929.s001.docx]

**Appendix**

**1. NN：neutral sentences**

（1）某人打电话叫工人来修理洗衣机

（2）某人在商场买了一些日用品

（3）某人拿着一些办公用品等公交车

（4）某人给自己准备好明天工作的材料

（5）某人帮邻居保管好日常生活用品

（6）某人在寝室卷起袖子准备洗衣服

（7）某人下班后乘坐出租车准备回家

（8）某人去小区周边水果店买水果

（9）某人上学前在家佩戴校徽

（10）某人放学后在地铁上听音乐

（11）某人下班后去超市买水果

（12）某人在书房用电脑制作PPT

（13）某人在放假期间和朋友一起逛街

（14）某人睡觉前在床上看推理小说

（15）某人从家里开车去超市买东西

（16）某人在厨房洗水果招待客人

（17）某人在办公室里签署文件

（18）某人在厨房洗手准备做午饭

（19）某人在家里起床后整理被褥

（20）某人出门前照镜子整理仪容

（21）某人在图书馆边看书边做笔记

（22）某人有空时上网浏览新闻

（23）某人周日在家里整理房间

（24）某人热身后去操场跑步

（25）某人下课后去文具店买本子

（26）某人吃完饭后去公园锻炼身体

（27）某人和家人去电影院看电影

（28）某人周末将新家具搬进新家

（29）某人植树节和同学在山上种树

（30）某人下雨时关上了教室的窗户

（31）某人早上骑自行车去公司上班

（32）某人吃完饭后在寝室睡午觉

（33）某人下班后网购电子产品

（34）某人和朋友讨论下阶段工作进程

（35）某人去眼镜店配了一副新眼镜

（36）某人在家洗澡后打电子游戏

（37）某人乘坐飞机去外地出差

（38）某人把便当带到公司当午餐

（39）某人在学校操场约同学见面

（40）某人在家里擦拭电器上的灰尘

（41）某人邀请朋友参观家中飞机模型

（42）某人下班后去银行办理信用卡

**2. MN：moral violation sentences that have nothing to do with physical impurity**

（1）某人捐款时把假币捐给灾区

（2）某人深夜大音量放音乐打扰他人

（3）某人故意欠朋友的钱不还

（4）某人故意告诉同学错误的考试时间

（5）某人在公众场所顺手拿别人东西

（6）某人在超市偷拿小物品

（7）某人在朋友家里偷拿一枚纪念币

（8）某人工作上收受他人的贿赂

（9）某人将拾到的他人财物占为己有

（10）某人在网络论坛里随意骂人

（11）某人踩到别人不道歉反而骂人

（12）某人大声辱骂自己的父母

（13）某人在马路上碰瓷索要赔偿金

（14）某人伪造学习和工作简历

（15）某人将自己弄伤以骗取保险

（16）某人把公共电话亭的电话线剪断

（17）某人毁坏公园垃圾箱等公共设施

（18）某人恶意拨打119火警电话

（19）某人在火车上将垃圾扔出窗外

（20）某人在火车站排长队买票时插队

（21）某人在他人遭遇危险时视而不见

（22）某人欺负年老残疾的乞讨者

（23）某人打听和传播他人的隐私

（24）某人在电影院时大声和朋友聊天

（25）某人在马路边将车乱停乱放

（26）某人不归还他人掉落的财物

（27）某人嘲笑身体上有残疾的人

（28）某人不尊重干脏活累活的人

（29）某人看到他人不幸时幸灾乐祸

（30）某人报复那些指出自己错误的人

（31）某人浪费公共场所的水电资源

（32）某人在公共场合讲粗话脏话

（33）某人借他人贵重物品不及时归还

（34）某人在马路旁抢乘出租车

（35）某人和亲友聚餐时铺张浪费

（36）某人路遇受伤者不予理睬

（37）某人在自习室里大声打电话

（38）某人故意误导前来问路的人

（39）某人把朋友的秘密说给其他人听

（40）某人挑拨朋友之间的关系

（41）某人用钥匙把别人的车刮花

（42）某人大声嘲笑他人的穿着

**3. MD：moral violation sentences that are related to physical impurity**

（1）某人带宠物任其随地大小便

（2）某人在公共游泳池里拉大便

（3）某人在公共游泳池里撒尿

（4）某人在步行街上拉大便

（5）某人将鼻涕擦到图书馆书架上

（6）某人把口水吐到图书馆的书上

（7）某人带小孩在公园草地大便

（8）某人在公厕上完厕所后不冲水

（9）某人故意把疾病传染给他人

（10）某人在公共场合脱鞋晾脚

（11）某人在公共场所随地吐痰

（12）某人把鼻屎粘到别人的书上

（13）某人故意对着别人的脸打喷嚏

（14）某人故意将脚气传给别人

（15）某人在公厕将小便尿到便池外

（16）某人故意用室友的洗脸巾擦脚

（17）某人故意用别人的牙刷刷马桶

（18）某人往别人饭菜里吐口水

（19）某人在楼上往楼下泼洗脚水

（20）某人用公用洗衣机洗内裤

（21）某人用地沟油炒菜给别人吃

（22）某人用腐败食材做饭给别人吃

（23）某人收集泔水制作地沟油

（24）某人往他人的水杯里吐痰

（25）某人随意乱扔用过的厕纸

（26）某人把鼻涕纸扔到别人碗里

（27）某人将发霉的面包拿给他人吃

（28）某人浑身汗臭还躺在别人的床上

（29）某人将食物残渣倒在他人身上

（30）某人故意把灰尘扬到别人碗里

（31）某人吃饭时把口水喷到别人碗里

（32）某人满头大汗试戴商店的帽子

（33）某人用酒店的电热水壶煮内裤

（34）某人将口香糖黏在公共座椅上

（35）某人将垃圾倒在领居家门口

（36）某人将鼻血擦在商场的新衣服上

（37）某人喝醉后将呕吐物吐到地铁上

（38）某人故意将别人推到臭水沟里

（39）某人故意开车溅他人一身脏水

（40）某人将手上的呕吐物抹到他人身上

（41）某人将剩饭剩菜倒到他人身上

（42）某人在公共场所乱吐瓜子皮
