# Supplementary material for: Different influences of moral violation with and without physical impurity on face processing: An event-related potentials study
Source: PLoS One. 2020 Dec 16;15(12):e0243929. doi: 10.1371/journal.pone.0243929 (PMC7743946; doi:10.1371/journal.pone.0243929)
Supplement: Attachment — Submitted filename: Response to Reviewers.docx [file pone.0243929.s004.docx]

Dear Dr. Peng,

Thank you for submitting your manuscript to PLOS ONE. After careful consideration, we feel that it has merit but does not fully meet PLOS ONE’s publication criteria as it currently stands. Therefore, we invite you to submit a revised version of the manuscript that addresses the points raised during the review process.

First, I greatly appreciate your detailed attention to the reviewers' initial comments and suggestions. The manuscript is much stronger. However, the interpretation of your results and the language used to explain your results need to be tempered. Given the design of the pilot study and the main study, it cannot be concluded that the moral violation sentences (with or without impurity) elicited or induced disgust. As you note in the limitations section, other emotions or alternative explanations cannot be ruled out. The description of your findings needs to be precise (e.g., moral violation sentences facilitated processing of disgust faces). Similarly, please make sure that the description of previous work in the introduction is precise. For example, around line 85, you conclude that moral violations activate disgust because of overlapping neural activity from disgust and moral violation stimuli. Despite the similar patterns of neural activity, we cannot conclude that disgust was activated, rather all we can say is that neural activity associated with disgust was observed with the presentation of moral violation stimuli. Other, different neural activity patterns were also observed. Please revise the manuscript tightening up the language throughout to more precisely describe results.

Author’s response:

Thank you very much for giving us constructive comments.

I strongly agree with the questions you raised. Experimental task we used in the study could not be completely achieved the previous purpose that we want to investigate whether WN also induced disgusted emotion. In the new version, the purpose of the study was changed to exploring the different neural mechanisms between WD and WN by using affective priming effect. The abstract was adjusted. At the same time, two fMRI studies cited were modified to make them more consistent with the original paper. In the discussion section, we describe the results more objectively and are cautious to infer the implication of results.

See Line 5-8, 16-17, 19, 76-81, 86-87, 89-91, 93, 110-111, 121-123, 384, 388-390, 399-401, 406-408, 423, 425, 433-434, 437-438, 445,
